# Management strategies for vestibular schwannomas in patients with NF2-related schwannomatosis: a systematic review

**DOI:** 10.1007/s00405-026-10536-x

**Published:** 2026-08-20

**Authors:** Helbert de Oliveira Manduca Palmiero, Eberval Gadelha Figueiredo

**Affiliations:** Division of Neurosurgery, University of São Paulo Medical School, São Paulo, Brazil

**Keywords:** NF2-related schwannomatosis, Vestibular schwannoma, Radiosurgery, Microsurgery, Systemic therapy

## Abstract

**Background:**

NF2-related schwannomatosis is a rare tumor-predisposition syndrome characterized by bilateral vestibular schwannomas (VS) that frequently lead to progressive hearing loss and neurological morbidity. Multi-modality strategies—microsurgery, radiosurgery, radiotherapy, systemic therapy, observation—are employed, but optimal treatment selection remains challenging due to disease heterogeneity, evolving practice patterns, and limited comparative evidence. This study assesses reported management strategies and outcomes for NF2-associated VS.

**Methods:**

A systematic review was performed for studies reporting management strategies and outcomes for NF2-associated VS, including cohort studies and case series describing treatment methods and outcomes. Data collected included treatment modalities, tumor control, hearing outcomes, and facial nerve function.

**Results:**

Of 359 records, 32 studies met the inclusion criteria, mostly retrospective case series. Radiosurgery represented the most frequently reported modality, followed by microsurgery. Radiosurgery achieved tumor-control rates ranging of 66%-100%, 5-year control rates of 81%-85%, and hearing preservation of 33%-67%. Microsurgical series generally involved larger tumors, with resection rates of 66%-92%, good facial nerve outcomes from 49.6% to 83.2%, and variable hearing preservation. Radiotherapy demonstrated tumor control of 87%-94%. In bevacizumab cohorts, radiographic response was observed in roughly one-third of tumors, stable disease in about half, and rebound growth after withdrawal in a subset.

**Conclusion:**

Management of NF2-associated VS remains individualized. Radiosurgery provides tumor control for selected tumors, but contemporary decision-making increasingly weighs long-term hearing goals, systemic options, and concern for radiation-associated malignant progression. Microsurgery prevails for larger or symptomatic lesions. Prospective studies with standardized outcomes and modern treatment comparisons are needed to refine treatment strategies.

**Graphical Abstract:**

**Figure:**
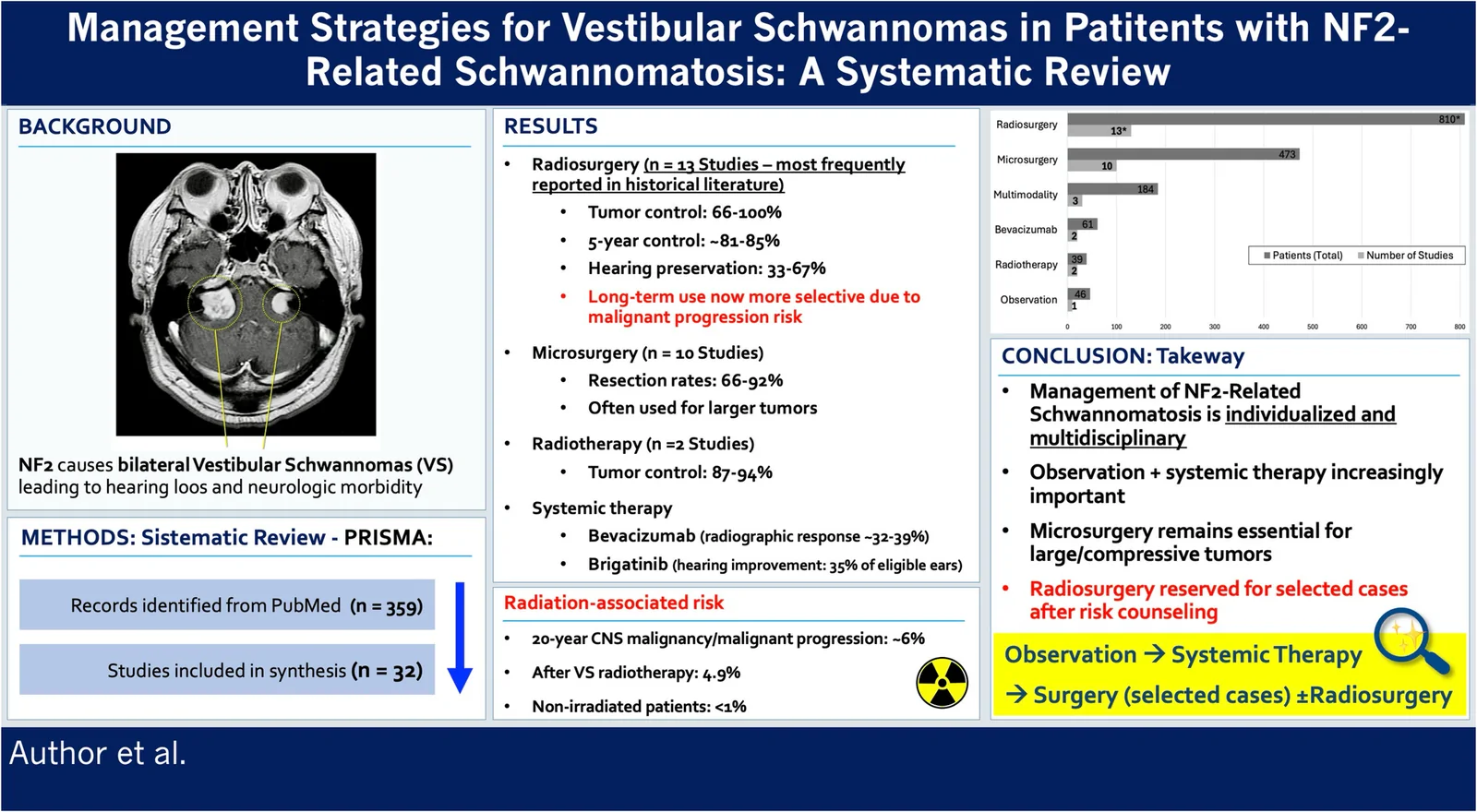

## Introduction

NF2-related schwannomatosis (NF2; formerly neurofibromatosis type 2) is a rare autosomal dominant tumor-predisposition syndrome caused by pathogenic mutations in the NF2 gene on chromosome 22q12. Updated international diagnostic criteria adopted in 2022 recognized the term NF2-related schwannomatosis, although the abbreviation NF2 remains widely used in the clinical literature [1]. The disorder is characterized by the development of multiple tumors in the central and peripheral nervous systems [2–4]. Bilateral vestibular schwannomas are the clinical hallmark of the disease, affecting approximately 90% to 95% of patients [2–7]. Although these tumors are histologically benign and often slow-growing, their clinical progression varies and may become progressive, with increasing morbidity mainly due to hearing loss, tinnitus, imbalance, brainstem compression in selected cases, and treatment-related cranial nerve dysfunction [2, 3, 5–7]. Since NF2-related vestibular schwannomas may demonstrate periods of quiescence or saltatory growth, initial monitoring with serial MRI and audiologic follow-up is reasonable in carefully selected patients. However, continued observation does not eliminate the risk of tumor growth or irreversible hearing deterioration, and many patients eventually require active treatment—especially in bilateral disease—raising important considerations regarding timing, side prioritization, and preservation of long-term neurological function [3, 5–7].

**Fig. 1:**
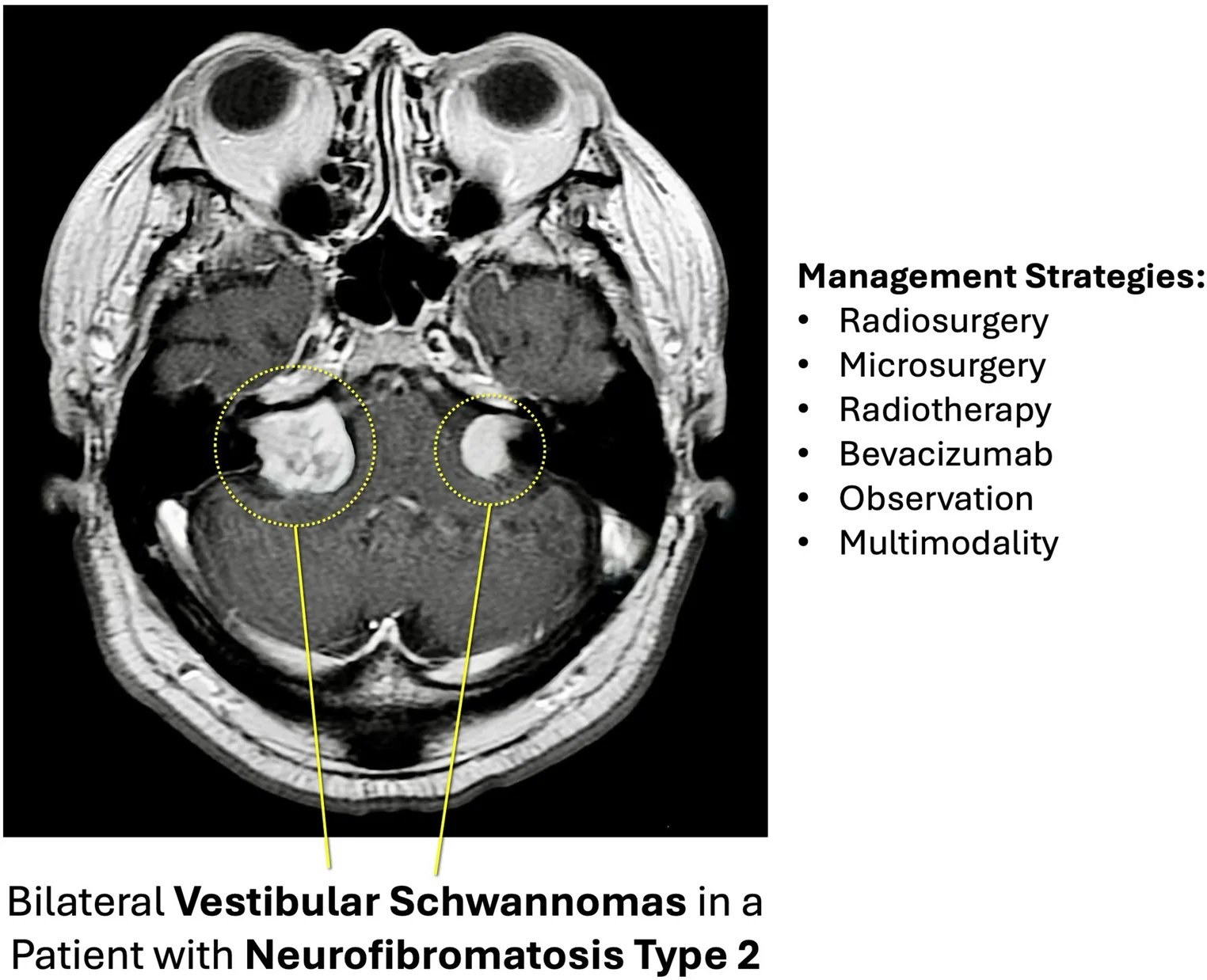
Axial contrast-enhanced MRI demonstrating bilateral vestibular schwannomas in a patient with NF2-related schwannomatosis. Bilateral vestibular schwannomas are considered characteristic of NF2 and occur in approximately 90-95% of affected patients. The lesions (yellow circles) originate from the vestibulocochlear nerves within the cerebellopontine angles. Available management strategies include radiosurgery, microsurgery, radiotherapy, bevacizumab (medical therapy), observation, and multimodal approaches

Current therapeutic options for NF2-associated vestibular schwannomas include microsurgical resection, stereotactic radiosurgery, fractionated radiotherapy, systemic therapy with bevacizumab in selected progressive cases, and multimodal treatment strategies tailored to tumor behavior, hearing status, age, and overall disease severity [4, 5, 8–11] (Fig. 1). More recently, brigatinib has emerged as a promising systemic option for progressive NF2-related schwannomatosis, with hearing improvement reported in 35% of eligible ears despite relatively modest volumetric response in vestibular schwannomas within a multi-tumor phase 2 platform trial [12]. Microsurgery remains a fundamental option, particularly for larger tumors, brainstem compression, or cases requiring decompression or cytoreduction, but it carries a substantial risk of hearing loss and facial nerve morbidity even at specialized centers [5, 9]. Radiosurgery has historically been an important alternative for small- to medium-sized tumors, providing effective tumor control with relatively low rates of trigeminal and facial nerve injury, though hearing preservation remains inconsistent, and concerns about delayed treatment failure and radiation-associated malignant progression now weigh more heavily in treatment selection [9–11, 13]. Meanwhile, bevacizumab has expanded treatment options for progressive schwannomas and has contributed to a shift toward personalized, function-oriented management in specialized multidisciplinary centers [3–5].

Given the rarity of NF2-related schwannomatosis, the heterogeneity of presentation, and the coexistence of multiple management strategies with distinct risk-benefit profiles, a comprehensive synthesis of the available evidence is clinically important. The purpose of this systematic review was to examine how vestibular schwannomas in patients with NF2 are currently managed, including the clinical circumstances prompting intervention, the therapeutic modalities employed, and the reported outcomes for tumor control, hearing preservation, and facial nerve function.

## Methods

This systematic review was conducted in accordance with the PRISMA 2020 guidelines [14].

### Search strategy and sources

A structured literature search of PubMed/MEDLINE was conducted from database inception through February 2026. The search was limited to studies published in English. Search terms included controlled vocabulary and free-text terms for NF2-related schwannomatosis (“*Neurofibromatosis 2*,” “*Neurofibromatosis type 2*,” “*NF2*,” “*NF2-related schwannomatosis*”), vestibular schwannoma (“*vestibular schwannoma*,” “*acoustic neuroma*,” “*acoustic neurinoma*,” “*vestibular neurinoma*”), and management approaches (“*microsurgery*,” “*surgery*,” “*radiosurgery*,” “*stereotactic radiosurgery*,” “*Gamma Knife*,” “*CyberKnife*,” “*LINAC*,” “*radiotherapy*,” “*bevacizumab*,” “*brigatinib*,” and “*treatment*”). Reference lists of the included studies were manually screened for additional eligible articles.

### Eligibility criteria

Studies were eligible if they reported management strategies or treatment outcomes for vestibular schwannomas in patients with NF2-related schwannomatosis. Cohort studies and case series were included. Studies were excluded if they focused on sporadic vestibular schwannomas, did not allow characterization of treatment modality or outcomes, or were case reports, narrative reviews, systematic reviews, or meta-analyses. No minimum sample-size threshold was applied: eligible cohort studies and case series were included irrespective of the number of patients or tumors reported, provided that the treatment modality and corresponding outcomes could be characterized. This inclusive approach was chosen deliberately because NF2-related schwannomatosis is rare and much of the informative evidence derives from small institutional series; imposing an arbitrary size cut-off would have excluded clinically relevant cohorts and biased the synthesis toward the few large centers. The trade-off of this decision is discussed in the limitations.

### Study selection

Two reviewers independently screened titles and abstracts, then reviewed the full texts of studies that appeared potentially eligible. Disagreements were settled through consensus. When overlapping cohorts were found, the dataset that was more complete or more recent was used for the descriptive synthesis.

### Data extraction and synthesis

Data were collected for study year, country/center, study design, number of patients and/or tumors, treatment modality, follow-up, pre-treatment symptoms, primary outcomes, and tumor control. Treatment modalities were categorized as radiosurgery, microsurgery, radiotherapy, medical therapy (bevacizumab), observation, or multimodal/combined management. Because of heterogeneity in study design, patient selection, outcome definitions, and reporting metrics, a descriptive qualitative synthesis was conducted. Results were compared across treatment modalities, focusing on tumor control, hearing results, facial nerve outcomes, and follow-up.

### PRISMA flow

**Fig. 2:**
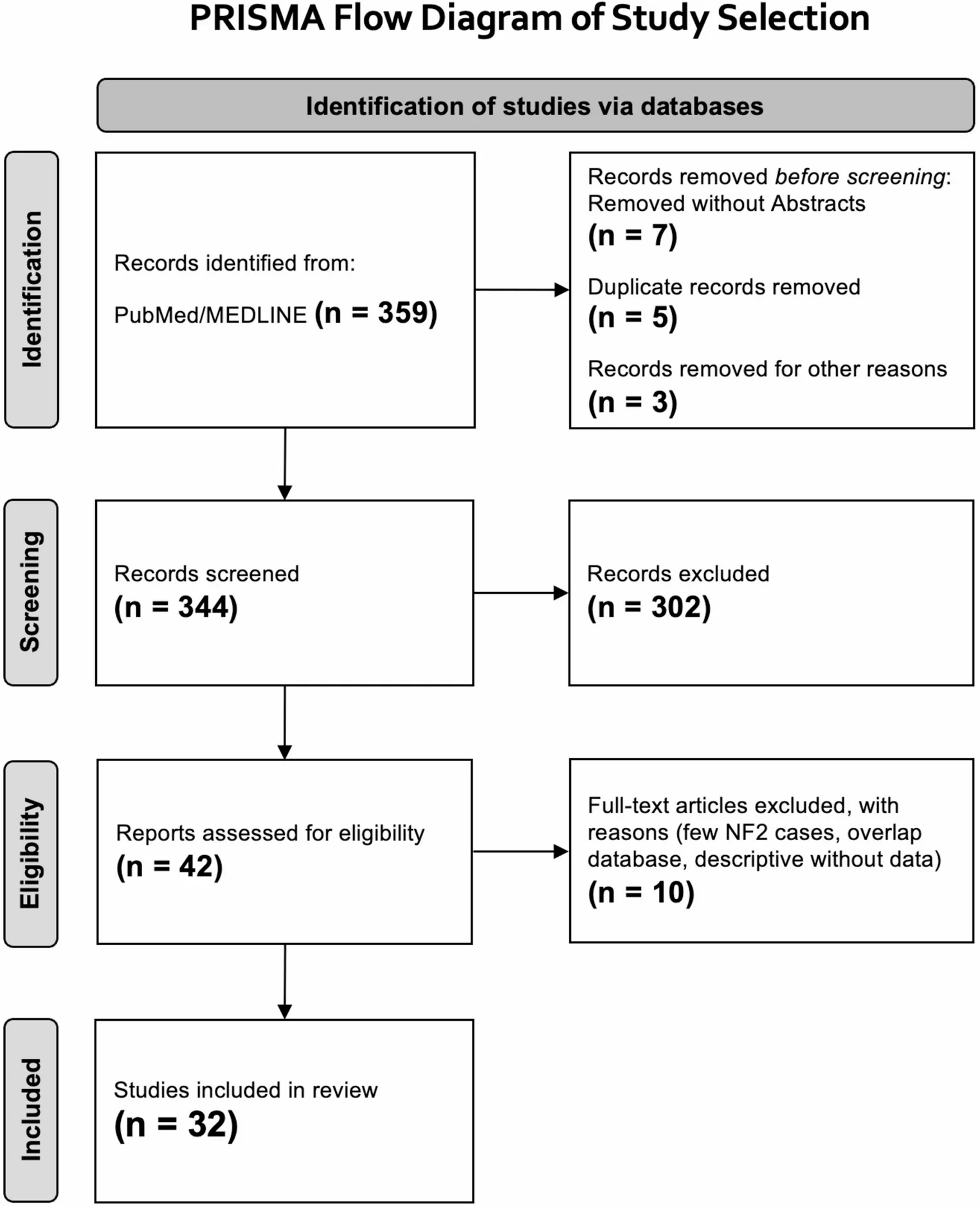
PRISMA 2020 flow diagram. Summarizing study identification, screening, eligibility, and inclusion for management strategies of vestibular schwannomas in patients with NF2-related schwannomatosis

The search identified 359 records. After screening and full-text review, 32 studies met the inclusion criteria and were included in the final analysis (Figure 2).

### Risk of bias assessment

Risk of bias was evaluated using the Newcastle-Ottawa Scale (NOS) for nonrandomized studies. The NOS assesses methodological quality across three domains: selection, comparability, and outcome assessment, with a maximum score of 9 points. Studies scoring 7–9 points were considered low risk of bias, 4–6 points as moderate risk, and ≤ 3 points as high risk. Disagreements were resolved through consensus.

### Ethics

Because this study relied solely on previously published data, institutional review board approval and informed consent were not necessary.

## Results

The literature search identified 359 records, of which 32 studies met the inclusion criteria after screening and full-text review (Fig. 2). The included evidence base was mainly composed of retrospective case series, with substantial variation in cohort size, treatment era, and outcome reporting. Most studies focused on radiosurgery or microsurgery, whereas radiotherapy, bevacizumab, observation, and multimodal treatment approaches were less frequently represented (Table 1).

**Table 1:** Characteristics and principal outcomes of included studies on the management of vestibular schwannomas in patients with NF2-related schwannomatosis. Patient and tumor counts are reported as provided in the original studies. Tumor control includes radiographic stability or regression unless otherwise specified by the source study. Follow-up is reported as mean or median per the original publication. Hearing outcomes were reported using study-specific definitions and scales, including AAO-HNS and Gardner-Robertson classifications when available

Study (Year)	Country/Center	Design	*n* (Patients/Tumors)	Treatment	Follow-up	Pre-Treatment Symptoms	Primary Outcomes	Tumor Control	Findings
Linskey (1992) [15]	USA (University of Pittsburgh, Presbyterian University Hospital)	Retrospective cohort	17/19	***Radiosurgery*** (Gamma Knife, multisource gamma unit)	Median imaging FU 1.4 y (0.3–3.9)	Pre-op hearing loss in some	Tumor control; tumor regression; hearing preservation; facial neuropathy	Tumor control 89.5%; regression 21.1% overall, 40% if ≥ 12 mo	Early landmark NF2 radiosurgery study with contralateral untreated controls; 17 patients unilateral SRS, 2 later treated bilaterally
Subach (1999) [16]	USA (University of Pittsburgh)	Retrospective cohort	40/45	***Radiosurgery*** (Gamma Knife)	Median 36 mo; subset median 92 mo for > 5-y follow-up	42/45 tumors with hearing loss; many prior surgeries	Tumor control; hearing; facial/trigeminal morbidity	Mean tumor volume 4.8 cm3	During median 36 mo, 36% regressed, 62% remained unchanged, and 2% grew; 3 patients (7%) later required surgery
Rowe (2003) [17]	UK (Royal Hallamshire Hospital, Sheffield)	Retrospective cohort	96/122	***Radiosurgery*** (Gamma Knife)	Estimated 8-y outcomes; subgroup mean 50 mo	Baseline hearing function assessed (Gardner-Robertson/PTA)	Tumor control; hearing; complications	Mean size 4.6 cm3; 8 y surgery-needed 20%; radiologic control 50%	At 8 y after radiosurgery, 20% had undergone surgery, 50% had radiologically controlled tumors, and 30% remained under conservative surveillance; 40% retained Gardner-Robertson hearing grade at 3 y
Mathieu (2007) [18]	USA/Canada (Pittsburgh-led multicenter/University of Sherbrooke)	Retrospective cohort	62/74	***Radiosurgery*** (Gamma Knife)	Median 53 mo	Serviceable hearing in 35% pre-SRS	Tumor control; hearing preservation; facial/trigeminal morbidity	Mean tumor volume 5.7 cm3; local control 85%/81%/81% at 5/10/15 y	Long-term control with modern lower-dose technique; hearing preservation improved with contemporary technique
Vachhani (2007) [19]	USA (University of Florida, Gainesville)	Retrospective cohort	13/14	***Radiosurgery*** (LINAC)	Average 38 mo	Bilateral VS in NF2; baseline symptom details not specified in abstract	Tumor control in treated and untreated sides	Treated tumors: actuarial local control 100% at 1 y and 92% at 2 and 5 y; untreated tumors 100%, 78%, and 21% at 1, 2, and 5 y	In all but 1 patient with follow-up data, radiosurgery prevented or reversed growth; approximately half of untreated contralateral tumors continued to grow
Meijer (2008) [20]	Netherlands (Vrije Universiteit University Medical Center, Amsterdam)	Retrospective cohort	25/25	***Radiosurgery*** (LINAC-based stereotactic)	Mean 51 mo (12–109 mo)	Tumor progression on serial MRI and/or progressive hearing loss; all untreated sides had hearing loss and 8/25 had ipsilateral deafness	Tumor control; minimal facial and trigeminal nerve toxicity; >40% retained hearing level or lost < 15 dB	Mean tumor diameter 2.5 cm	Five patients had < 1-y follow-up; in the remaining 20, local control was 100% with no trigeminal/facial toxicity. Mean PTA worsened from 51 to 77 dB; no additional hearing loss occurred beyond 36 mo
Rowe (2008) [21]	UK (Royal Hallamshire Hospital, Sheffield)	Retrospective cohort	96 (+ 22)/122(+ 24)	***Radiosurgery*** (Gamma Knife)	Extended series 906 patient-years	Functional hearing preservation was a major baseline concern	Tumor control; hearing; malignancy risk	Tumors < 10 cm3 (good clinical outcomes), 94–96% of patients	In the 122-tumor/96-patient Sheffield series +extended 22 patients and 24 tumors, 8-y estimates were 20% needing further treatment, 50% well controlled, and 30% observed despite concern; ~40% retained functional hearing at 3 y and facial palsy risk was 5%; “tumor volume at the time of treatment is the most significant determinant of outcome”
Samii (2008) [22]	Germany (International Neuroscience Institute, Hannover)	Retrospective cohort	145/195	**Microsurgery**	Long-term follow-up examinations	24% deaf pre-op; unilateral useful hearing 34%; bilateral useful hearing 42%	Extent of resection; hearing preservation; facial nerve preservation	85% total removal; hearing preserved overall in 35%; facial nerve integrity 89%	Among 145 patients/195 surgeries, 85% underwent total removal and 15% deliberate subtotal resection for brainstem decompression or hearing preservation; hearing preservation was 35% overall (65% among those with useful pre-op hearing), bilateral hearing 23%, and facial nerve integrity 89%
Phi (2009) [23]	South Korea (Seoul National University)	Retrospective cohort	30/36	***Radiosurgery*** (Gamma Knife)	Median clinical 48.5 mo; radiologic 36.5 mo	Ipsilateral serviceable hearing in 16 tumors before SRS	Tumor control; serviceable hearing; salvage surgery	Median tumor volume 3.2 cm3; actuarial control 81%/74%/66% at 1/2/5 y	Low marginal dose and young age predicted poorer control
Sharma (2010) [24]	India (AIIMS, New Delhi)	Retrospective cohort	30/54	***Radiosurgery*** (Gamma Knife Leksell model B)	Average 26.6 mo clinical; 14 radiologic patients	Hearing loss common presentation	Tumor control; hearing preservation; facial nerve preservation	Average tumor size 3.7 cc; control 87.5%; hearing preservation 66.7%	Early Indian GKRS NF2 series
Odat (2011) [25]	Italy (Gruppo Otologico, Piacenza)	Retrospective cohort	38/48	**Microsurgery**	Mean 3.7 y	Symptoms often included tinnitus, gradual hearing loss, and vestibular dysfunction; larger tumors risked brainstem compression, hydrocephalus, and facial palsy	Hearing rehabilitation; facial nerve preservation	Average tumor size 3.1 cm	Across 48 operations in 38 NF2 patients, total resection was achieved in 92%; postoperative House-Brackmann grade I-III facial function occurred in 77% of cases
Tysome (2012) [26]	UK (Cambridge NF2 Unit)	Retrospective cohort	75/58	**Microsurgery** + Hearing rehabilitation (ABI/cochlear implant)	1981–2011 cohort; 12-mo postoperative facial outcomes reported	Hearing loss was the dominant quality-of-life issue; 72% of current patients had AAO-HNS A–C hearing in the better ear	Tumor size; hearing and facial function; auditory implantation outcomes	44 patients underwent resection of 50 VS; 43 untreated VS were ≤ 20 mm and stable for ≥ 5 y	Fifty-eight current patients were reviewed; 44 underwent 50 resections. At 12 mo, facial outcomes were HB1 33%, HB2 21%, HB3 30%; total resection was achieved in 78%, and proactive cochlear implant/auditory brainstem implant rehabilitation preserved auditory function in many current patients
Moffat (2013) [27]	UK (Cambridge & Manchester NF2 units)	Retrospective cohort	128/148	**Microsurgery**	1–30 y; mean 12.4 y	Preoperative tinnitus present in 73%; hydrocephalus in 26%; some had prior failed stereotactic RT	Extent of resection; facial nerve outcomes; recurrence	Mean tumor size 3.1 cm; total excision 66%; residual/recurrence 13.9%	Among 148 translabyrinthine operations, mean tumor size was 3.1 cm, residual/recurrence was 13.9%, perioperative mortality 1.6%, and HB I–III facial function at 2 y was 83.2%; 56 patients received ABI and 2 had CI sleepers
Peyre (2013) [28]	France (Paris)	Retrospective cohort	46/92	Observation (Conservative Management)	Mean clinical 6.0 y; mean radiologic 4.2 y	Serviceable hearing at baseline; small tumors	Tumor growth; surgery-free survival; hearing preservation	Mean tumor diameter 13 mm baseline, 20 mm final; mean growth 1.8 mm/y	Observation feasible in selected small/serviceable-hearing tumors; no correlation between growth and hearing preservation
Mallory (2014) [29]	USA (Mayo Clinic, Rochester)	Retrospective cohort	26/32	***Radiosurgery*** (Gamma Knife, single-session, stereotatic)	Median 7.6 y	Baseline hearing class and facial nerve function were recorded; symptom spectrum not separately detailed in abstract	Progression-free survival; hearing; facial nerve preservation	Median tumor volume 2.7 cm3; 5y PFS 85%; 10y PFS 80%	Of 32 tumors, 84% showed no growth at median 7.6-y follow-up; 5- and 10-y PFS were 85% and 80%. Higher marginal dose improved control, but hearing outcomes remained poor despite contemporary reduced dosing
Wagner (2014) [30]	Germany (Heidelberg and Technical University Munich)	Retrospective cohort	14/16	Radiotherapy (FSRT) or ***Radiosurgery*** (SRS)	Median 129 mo (39–229)	Patients were selected for treatment because of tumor progression and/or progression of clinical symptoms	Tumor control; hearing; facial/trigeminal toxicity	Mean tumor size 3 cm; local control 94%; hearing preservation 33%	Sixteen VS in 14 NF2 patients were treated (14 FSRT, 2 SRS). Local control was 94% at median 131 mo, pretreatment hearing level was maintained in 44%, useful hearing in 33%, and cranial nerve toxicity remained moderate
Kim (2016) [31]	South Korea (Samsung Medical Center/Seoul)	Retrospective cohort	17/32	Mixed Management: **microsurgery**; ***radiosurgery*** (fractionated GKRS; single-session GKRS); conservative	MS 3.9 y; f-GKS 5.1 y; s-GKS 8.4 y; CM 6.1 y	Hearing deterioration	Tumor volume change; hearing status by initial modality	Mean tumor volume: MS 1.6 mL; f-GKS 11.1 mL; s-GKS 5.6 mL; CM 1.6 mL	Multi-modality institutional series comparing first-line strategies by ear
Morris (2016) [32]	UK national NF2 service (Oxford/Cambridge/Manchester etc.)	Prospective cohort	61/47	Bevacizumab	Median 15 mo (on-drug partial response)	Hearing loss; growing tumors	Radiologic response; hearing response; toxicity	Baseline index tumor volume 7.26 cc; best index response: 32% PR, 51% SD, 17% PD	National bevacizumab program showing meaningful but not universal volumetric response
Chen (2018) [33]	China (PLA Army General Hospital, Beijing)	Retrospective case series	17/26	**Microsurgery**	Mean 39 mo (range 11–78)	Progressive hearing loss; 7 ears deaf	Hearing preservation; facial nerve preservation; complications	26 VS excisions; hearing preservation 41.6%; facial nerve preservation 60%	Staged bilateral microsurgery reported with no mortality/major complication
Spatola (2018) [34]	France/Italy (Marseille database)	Retrospective cohort	103/129	***Radiosurgery*** (Gamma Knife)	Median 5.9 y	Serviceable hearing in 70 ears at GKRS	Progression-free survival; hearing; facial/trigeminal toxicity	Median tumor volume 1.5 cm3; median reduction 43% among shrunken tumors; 5y/10y PFS 88%/75%	Long-term single-center database with volumetric assessment
Zhao (2018) [35]	China (Beijing Tiantan Hospital)	Retrospective cohort	140/149	**Microsurgery**	2006–2016 cohort period	Large vestibular schwannomas (> 2 cm) with focus on preserving preoperative hearing and facial nerve function	Hearing preservation; facial nerve function	Large-size VS > 2 cm; 149 resections in 140 NF2 patients	Across 149 resections in 140 NF2 patients, total removal was achieved in 82.6%, facial nerve integrity in 67.8%, good facial outcome (HB I–III) at 12 mo in 49.6%, and class B/C hearing was preserved in 7/31 attempted hearing-preservation cases
Gugel (2019) [36]	Germany (University Hospital Tübingen)	Retrospective cohort	23/39	**Microsurgery**	21–167 mo	Hearing preservation focus	Hearing preservation longitudinally	39 operated tumors; 20 non-operated ears comparator; immediate hearing maintained in 82%	Retrosigmoid, BAEP-guided, microsurgery did not accelerate long-term hearing deterioration compared with non-operated ears
Gugel (2020) [37]	Germany (University Hospital Tübingen)	Retrospective cohort	28/46	**Microsurgery** ± Bevacizumab (Postoperative)	Minimum 12 mo	Hearing loss; tumor growth	Tumor volume and growth rate; hearing preservation	46 operated tumors; surgery reduced postoperative growth; 16 operated VS received bevacizumab	Surgery slowed growth; truncating mutations associated with faster postoperative growth
Przepiorka (2020) [38]	Poland (Medical University of Warsaw)	Retrospective case series	7 (in 29 VS patients)/29	**Microsurgery**	Mean 3.46 y (range 0.25–11; median 2)	Unilateral hearing loss; many with facial paresis	Extent of resection; facial outcomes	Mean recurrent tumor size 23.3 mm	Revision GTR was achieved in all 29 recurrent/remnant VS cases. Mean recurrent size was 23.3 mm; 7 patients had NF2; no tumors recurred during follow-up and facial neurorrhaphy improved severe paresis in selected cases
Troude (2020) [39]	France (Marseille single center)	Retrospective cohort	12 (vs. 69 non-NF2)/24	**Microsurgery** + ***Radiosurgery*** (Gamma Knife, Postoperative)	Mean 73 mo	Large tumors with facial-nerve sparing intent	Tumor control; facial nerve function	Median residual volume 0.92 cc in NF2	This comparative series included 12 NF2 and 69 non-NF2 patients with large VS treated by resection plus upfront Gamma Knife to the residue. In NF2, good facial function (HB I–II) was 92%, median residual volume 0.92 cc, and tumor control 83%; outcomes were similar to non-NF2 cases
Goshtasbi (2021) [40]	USA (National Cancer Database)	Retrospective cohort	155/Bilateral	Mixed Management: observation, **microsurgery**, radiotherapy, chemotherapy	Mean 67.3 mo	Clinical presentation not granularly coded in NCDB; bilateral VS/NF2-associated cohort identified by laterality	Treatment distribution; overall survival	Mean tumor size 23.5 mm	Among 155 bilateral VS/NF2-associated cases in the NCDB, observation was used in 45.3%, surgery 29.3%, radiotherapy 20.0%, chemotherapy 3.3%; 5-year mortality was 5.8%. Surgery was associated with larger tumors and radiotherapy with older age
Kim (2022) [41]	South Korea (Asan Medical Center, Seoul)	Retrospective case series	33/41	***Radiosurgery*** (Gamma Knife)	Median 69.1 mo (45.0–104.8)	Serviceable hearing status and treatment-related morbidities were assessed at baseline	Tumor volume response; hearing preservation; morbidity	Most treated lesions showed effective tumor control up to 85% at 60 mo; 29% showed early pseudoprogression; untreated lesions increased 14.0%/y	In 33 patients with 41 treated lesions, SRS achieved tumor control in most lesions. Pseudoprogression was common early (29%), and among patients with serviceable hearing, 2/10 (20%) lost hearing within 24 mo
Bin-Alamer (2023) [42]	International multicenter (IRRF; UPMC-led)	Retrospective case series	267/328	***Radiosurgery*** (single-session, multiple centers, mainly Gamma Knife)	Median 59 mo (IQR 23–112)	Hearing loss; cranial neuropathies common at baseline	Tumor control; freedom from additional treatment; serviceable hearing; HB/CN V/tinnitus/vestibulopathy	Median tumor volume 2.8 cc; 10y control 77%; 15y 52%; 26.2% decreased; 55.5% stable	Largest contemporary multicenter NF2 SRS series; no radiation-induced tumor or malignant transformation observed
Krishna (2023) [43]	India (AIIMS, New Delhi)	Retrospective cohort	85/133	***Radiosurgery*** (Gamma Knife)	Minimum 1 y; reported to 9 y	Typical NF2-VS symptom burden included tinnitus, balance dysfunction, and progressive hearing loss	Tumor control; hearing preservation; facial nerve preservation; retreatment	57 regressed, 35 stable, 23 progressed; overall control 85%	Among 133 tumors in 85 patients, 57 regressed, 35 were stable, and 23 progressed. Overall control was 85%; actuarial control was 95%, 79%, 75%, and 55% at 1, 3, 5, and 9 y. Five tumors required repeat GKRS
Puataweepong (2023) [44]	Thailand (Ramathibodi Hospital, Mahidol University, Bangkok)	Retrospective cohort	25/48	Radiotherapy (Robotic hypofractionated stereotactic, CyberKnife HSRT)	Median 98 mo (24–155)	Patients had bilateral NF2-VS selected for HSRT	Local control; hearing preservation; non-auditory complications	Median tumor volume 2.3 cm3; 5y/10y control 87%/80%; hearing preservation 59%/35%	In 25 NF2 patients with 48 VSs treated with robotic HSRT, median tumor volume was 2.3 cm3 and median follow-up 98 mo. Local control was 87%/80% at 5/10 y and hearing preservation 59%/35%; three patients developed transient-volume-expansion symptoms (hydrocephalus, facial weakness, ataxia)
Webb (2023) [45]	USA (Mayo Clinic, Rochester)	Retrospective cohort	19/15	Bevacizumab (Withdrawal)	Median bevacizumab duration 12.2 mo in discontinuation group; rebound assessed within 6 mo after stopping	VS growth and symptom progression under bevacizumab surveillance; hearing preservation was a treatment objective	Rebound tumor growth after stopping bevacizumab	15 evaluable brain MRIs: 5 PR, 8 SD, 2 PD before withdrawal; 5/8 with > 20% rebound growth within 6 mo	Of 19 treated patients, 13 stopped bevacizumab and 6 remained on treatment. Among 15 with evaluable MRI, 5 had partial response, 8 stable disease, and 2 progression; after discontinuation, 5/8 evaluable patients had > 20% rebound growth within 6 mo and 3 required surgery or irradiation
Hudelist (2025) [46]	International multicenter (Paris, Manchester, Mayo, NYU)	Retrospective cohort	21/21	**Microsurgery** (salvage after failed Bevacizumab)	Exact postoperative follow-up not specified in reviewed text; BEV stopped mean 5.8 ± 4.0 mo before surgery	Progression despite bevacizumab; surgery in NF2-SWN VS carries increased risks of hearing loss and facial nerve damage	Salvage surgery outcomes	Mean VS volume 13.2 ± 7.6 cm3 (1 Koos III, 20 Koos IV); mean residual volume 1.3 ± 2.0 cm3 after surgery	In this 4-center salvage microsurgery series after bevacizumab failure, higher cumulative BEV dose and later discontinuation predicted more intraoperative bleeding and longer surgery. Recommended BEV-free window before surgery: ~6 weeks to 6 months

*AAO-HNS* American Academy of Otolaryngology–Head and Neck Surgery; *ABI* auditory brainstem implant; *BAEP* brainstem auditory evoked potentials; *BEV *bevacizumab; *CI*cochlear implant; *CM *conservative management; *FSRT* fractionated stereotactic radiotherapy; *GKRS/GKS* Gamma Knife radiosurgery; *HB *House-Brackmann; *HSRT *hypofractionated stereotactic radiotherapy; *LINAC *linear accelerator; PFS progression-free survival; PTA pure-tone average; *SRS* stereotactic radiosurgery; *VS* vestibular schwannoma

Marked heterogeneity in patient selection, baseline hearing status, tumor size, follow-up duration, and outcome definitions precluded quantitative pooling. Radiosurgery was generally used for small- to medium-sized tumors and achieved tumor control rates of 66% to 100%, with long-term actuarial control around 81%-85% at 5 years in several series. Hearing preservation commonly ranged from 33% to 67%, whereas facial/trigeminal morbidity was usually low. Microsurgical cohorts more often included larger tumors or those associated with brainstem compression or progressive neurological compromise. Across microsurgical studies, gross-total or near-total resection ranged from 66% to 92%, hearing preservation was variable, facial nerve integrity ranged from 67.8% to 89%, and good facial nerve outcomes (House-Brackmann grade I-III) ranged from 49.6% to 83.2%, where reported.

Microsurgical cohorts more often included larger tumors, progressive hearing decline, mass effect, hydrocephalus, or concern for facial nerve dysfunction. In these studies, total resection ranged from 66% to 92%, and hearing preservation varied widely by tumor size and baseline hearing. By contrast, the limited radiotherapy literature reported local control rates of 87% to 94%, with long-term hearing preservation declining in some series.

Observation was described in a single study limited to selected small tumors with serviceable hearing at baseline, in which the mean tumor diameter increased from 13 to 20 mm at follow-up, despite generally favorable short- to intermediate-term hearing preservation. Medical therapy with bevacizumab was reported in two studies and was typically used for growing tumors accompanied by progressive hearing decline. In the national cohort, the best index radiographic response was 32% partial response, 51% stable disease, and 17% progression; an overall radiologic response rate of 39% has also been reported for index and non-index tumors in that series, and rebound growth of more than 20% within 6 months of bevacizumab withdrawal occurred in 5 of 8 evaluable patients [32, 45] 

**Fig. 3:**
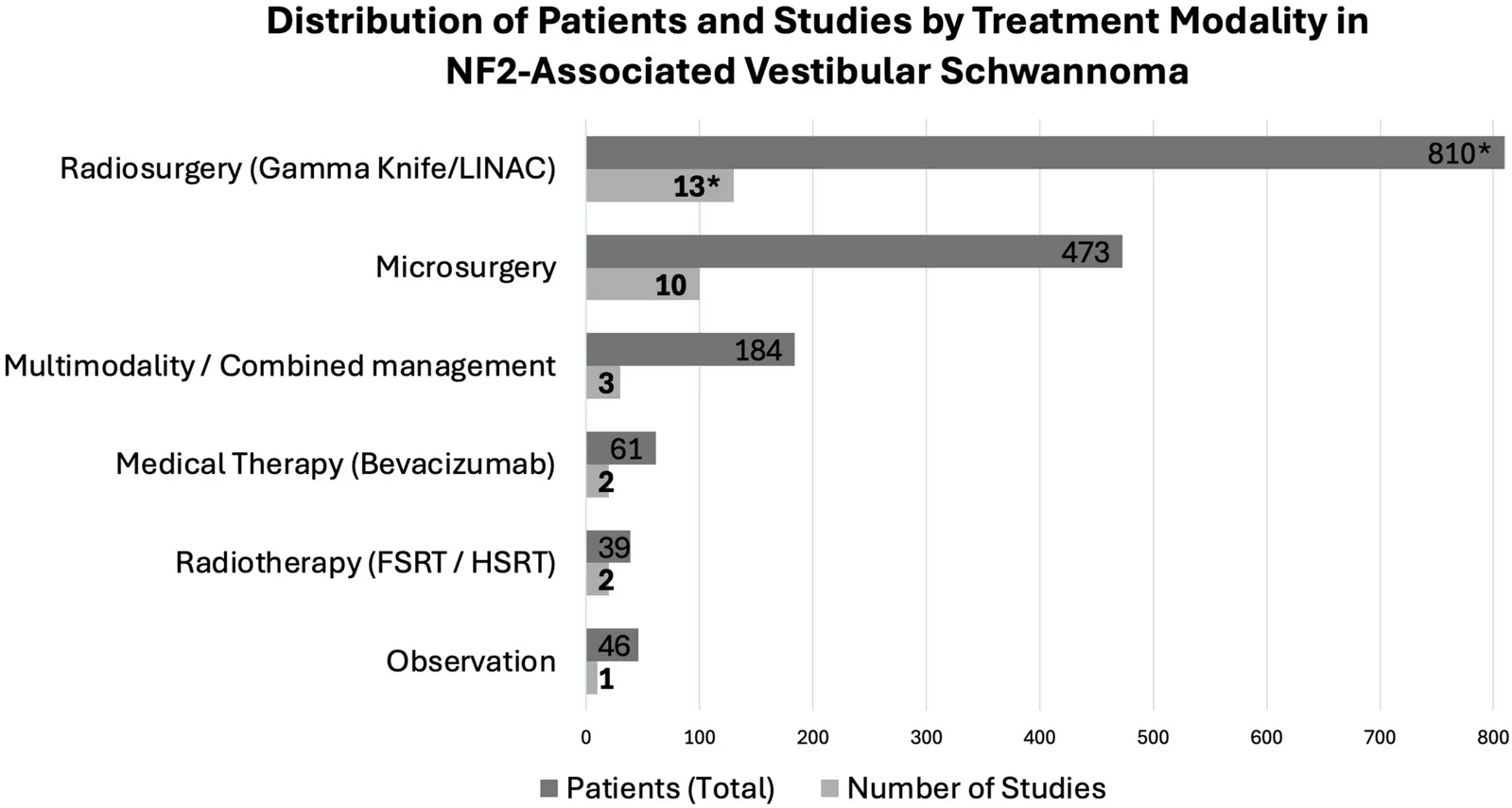
Distribution of included studies and total patients by treatment modality for vestibular schwannomas in patients with NF2-related schwannomatosis. Bars represent the total number of patients (dark gray) and the number of studies (light gray) providing data for each treatment method, including radiosurgery (Gamma Knife/LINAC), microsurgery, multimodality/combined management, medical therapy (bevacizumab), radiotherapy (FSRT/HSRT), and observation. *For the radiosurgery category, 13 studies and 810 patients are included in this figure. One study (Rowe 2003 [17]) was excluded from the graphical count because its cohort partly overlaps with a later expanded series from the same center (Rowe 2008 [21]). To prevent double-counting, the earlier study with fewer patients was removed

By treatment category, Table 2 lists 14 radiosurgery studies, 10 microsurgery studies, 3 multimodality studies, 2 radiotherapy studies, 2 bevacizumab studies, and 1 observational study. In Fig. 3, radiosurgery is the most frequently reported modality in the published literature; however, this pattern should be interpreted as reflecting the historical evidence base rather than serving as a direct proxy for current first-line practice.

**Table 2:** Treatment-modality summary of outcomes for bilateral vestibular schwannomas in patients with NF2-related schwannomatosis. Values indicate ranges or representative results from the included studies rather than pooled estimates due to heterogeneity in study design, patient populations, tumor size metrics, and outcome definitions. Tumor control refers to radiographic stability or regression unless specified otherwise in the original publication. Hearing outcomes were reported using various study-specific classifications, commonly Gardner–Robertson or AAO-HNS criteria. Facial nerve outcomes were generally reported with the House–Brackmann grading system. Follow-up duration is presented as mean or median based on the original studies. Treatment categories were defined according to the primary management modality

Treatment Modality	Studies (*n*)	Pre-treatment	Tumor control/growth	Hearing	Facial nerve	Follow-up
Radiosurgery (Gamma Knife/LINAC) [15–21, 23, 24, 29, 34, 41–43]	14	Typically small-to-moderate tumors; many cohorts selected progressive tumors and/or ears with residual serviceable hearing	Tumor control ranged from 66% to 100% depending on series, technique, and follow-up; long-term control was about 81%−85% at 5 years and 75%−81% at 10–15 years in some cohorts	Serviceable hearing preservation was commonly 33%−67%; some contemporary series still showed poor long-term functional hearing despite tumor control	Facial and trigeminal morbidity was generally low; facial worsening was about 5%−10% in larger series and permanent facial palsy was uncommon	26.6 mo to 8 y+ (actuarial outcomes reported to 9–15 y in some cohorts)
Microsurgery [22, 25–27, 33, 35–38, 46]	10	Usually larger tumors; progressive hearing loss common; some series included mass effect, hydrocephalus, or facial dysfunction risk	Total removal 66%−92%; residual/recurrence 13.9% in a long-term translabyrinthine series (Moffat [27]); surgery reduced postoperative growth in 1 pediatric/young-adult cohort (Gugel [37])	Hearing preservation 35% overall in Samii (Samii [22]), 41.6% in Chen (Chen [33]), and 82% immediate postoperative maintenance in Gugel (Gugel [36]); preservation in large tumors remained limited	Facial nerve integrity 67.8%−89%; good HB I-III outcome 49.6%−83.2% where reported	39 mo to 12.4 y (some cohorts spanned longer treatment eras)
Multimodality/ combined management [31, 39, 40]	3	Individualized treatment in specialized centers, including surgery plus upfront postoperative radiosurgery or mixed first-line strategies by ear	Combined resection plus upfront Gamma Knife for large tumors achieved 83% control in the NF2 subgroup of the comparative French series (Troude [39])	Hearing outcomes were heterogeneous and depended on baseline hearing and modality sequence	Good facial function (HB I-II) reached 92% in the combined surgery plus Gamma Knife NF2 subgroup (Troude [39])	Mean 73 mo in the combined-surgery/radiosurgery series; other cohorts reported modality-specific follow-up
Radiotherapy (FSRT/HSRT) [30, 44]	2	Progressive tumors and/or symptomatic bilateral disease; baseline hearing often already impaired	Local control 87%−94%	Hearing preservation 33%−59%; useful hearing tended to decline over long-term follow-up	Cranial nerve toxicity was moderate overall; transient volume-expansion symptoms occurred in 1 HSRT cohort (Puataweepong [44])	98 to 129 mo median follow-up
Medical therapy (Bevacizumab) [32, 45]	2	Growing tumors with progressive hearing decline were typical indications	In the national cohort, best index response was 32% partial response, 51% stable disease, and 17% progression (Morris [32]); after withdrawal, rebound growth > 20% within 6 mo occurred in 5/8 evaluable patients	Hearing response occurred in a subset during treatment, but durability after withdrawal was uncertain	Systemic toxicity varied; discontinuation could complicate later surgery and was associated with rebound growth in some patients	About 12–15 mo treatment follow-up
Observation [28]	1	Small tumors with serviceable hearing at baseline	Mean diameter increased from 13 mm to 20 mm; mean growth 1.8 mm/y	High serviceable-hearing preservation in selected small tumors; no clear correlation between growth and hearing preservation in the observational cohort	Not applicable as a primary endpoint	Mean clinical follow-up 6.0 y; mean radiologic follow-up 4.2 y

*FSRT* fractionated stereotactic radiotherapy; *HB* House-Brackmann; *HSRT* hypofractionated stereotactic radiotherapy; *LINAC* linear accelerator; *NF2* neurofibromatosis type 2; *SRS* stereotactic radiosurgery

### Risk-of-bias assessment using the new-ottawa scale

Demonstrated predominantly moderate methodological quality (Table 3). Out of the 32 included studies, 30 were rated as having a moderate risk of bias, 1 as low risk, and 1 as high risk. The single low-risk study was the recent international multicenter radiosurgery series by Bin-Alamer et al. [42], while the high-risk study was the small revision-surgery case series by Przepiorka et al. [38]. The predominance of moderate risk reflected the retrospective design of most studies, the absence of control groups, and inconsistent reporting of outcomes across treatment modalities.

**Table 3:** Risk-of-bias assessment of the included studies using the Newcastle–Ottawa Scale (NOS). Scores indicate the NOS for nonrandomized studies. Selection domain maximum = 4 points; Comparability = 2 points; Outcome = 3 points. Risk categories: *low risk* (7–9 points), *moderate risk* (4–6 points), *high risk* (≤ 3 points)

Study (Year)	Selection (0–4)	Comparability (0–2)	Outcome (0–3)	Total (0–9)	Risk category
Linskey (1992) [15]	3	1	2	**6**	**Moderate**
Subach (1999) [16]	3	1	2	**6**	**Moderate**
Rowe (2003) [17]	3	1	2	**6**	**Moderate**
Mathieu (2007) [18]	3	1	2	**6**	**Moderate**
Vachhani (2007) [19]	3	0	2	**5**	**Moderate**
Meijer (2008) [20]	3	0	2	**5**	**Moderate**
Rowe (2008) [21]	3	1	2	**6**	**Moderate**
Samii (2008) [22]	3	1	2	**6**	**Moderate**
Phi (2009) [23]	3	0	2	**5**	**Moderate**
Sharma (2010) [24]	3	0	2	**5**	**Moderate**
Odat (2011) [25]	3	0	2	**5**	**Moderate**
Tysome (2012) [26]	3	1	2	**6**	**Moderate**
Moffat (2013) [27]	3	1	2	**6**	**Moderate**
Peyre (2013) [28]	3	0	2	**5**	**Moderate**
Mallory (2014) [29]	3	1	2	**6**	**Moderate**
Wagner (2014) [30]	3	0	2	**5**	**Moderate**
Kim (2016) [31]	3	1	2	**6**	**Moderate**
Morris (2016) [32]	3	1	2	**6**	**Moderate**
Chen (2018) [33]	2	0	2	**4**	**Moderate**
Spatola (2018) [34]	3	1	2	**6**	**Moderate**
Zhao (2018) [35]	3	1	2	**6**	**Moderate**
Gugel (2019) [36]	3	1	2	**6**	**Moderate**
Gugel (2020) [37]	3	1	2	**6**	**Moderate**
Przepiorka (2020) [38]	2	0	1	**3**	**High**
Troude (2020) [39]	3	1	2	**6**	**Moderate**
Goshtasbi (2021) [40]	3	1	2	**6**	**Moderate**
Kim (2022) [41]	3	0	2	**5**	**Moderate**
Bin‑Alamer (2023) [42]	4	1	2	**7**	**Low**
Krishna (2023) [43]	3	1	2	**6**	**Moderate**
Puataweepong (2023) [44]	3	0	2	**5**	**Moderate**
Webb (2023) [45]	3	1	2	**6**	**Moderate**
Hudelist (2025) [46]	3	1	2	**6**	**Moderate**

## Discussion

This systematic review synthesizes the available evidence on management strategies for vestibular schwannomas in patients with NF2-related schwannomatosis (NF2). Across the 32 included studies, radiosurgery was the most frequently reported treatment, followed by microsurgery. However, this pattern primarily reflects the composition of the published literature—much of it derived from radiosurgery-focused cohorts from earlier treatment eras—rather than a definitive statement that radiosurgery is the dominant contemporary strategy across specialist NF2 programs.

Radiosurgery demonstrated favorable tumor control in most included studies, with control rates ranging from approximately 66% to 100% and long-term control rates around 75%-85% in several series. These outcomes support the efficacy of radiation-based local treatment for selected small- to medium-sized tumors. Nevertheless, interpreting these data now requires greater caution. A national UK study of 266 irradiated patients with NF2-related schwannomatosis found a cumulative 20-year risk of central nervous system malignancy or malignant progression of approximately 6% overall and 4.9% after vestibular schwannoma radiotherapy, compared with less than 1% in non-irradiated patients. The authors concluded that radiotherapy should not be offered as first-line treatment for benign tumors in this population [13]. This evidence helps explain why many contemporary centers have become more selective in their use of radiosurgery despite the relatively large historical radiosurgery literature.

Microsurgical resection remains an essential treatment modality, particularly for larger tumors, those causing brainstem compression, or cases in which decompression is necessary. The reviewed microsurgical series reported resection rates of 66% to 92%, with variable hearing outcomes that were generally better in smaller tumors and in carefully selected hearing-preservation procedures. Facial nerve preservation remained a key benchmark, with facial nerve integrity ranging from 67.8% to 89% and House-Brackmann grade I-III outcomes ranging from 49.6% to 83.2% where reported.

Radiotherapy with fractionated stereotactic techniques has been reported in only a few studies, but reported tumor control rates were generally high, ranging from 87% to 94%. Even so, the same long-term concerns that now affect stereotactic radiosurgery counseling—especially the possibility of radiation-associated malignant progression in a tumor-predisposition syndrome—should be incorporated into treatment discussions [13].

Observation remains a feasible strategy for carefully selected patients with small tumors and preserved hearing. The limited observational data in this review confirm that some NF2-associated vestibular schwannomas may remain indolent for prolonged periods, whereas others continue to grow despite an initial surveillance period. This supports MRI- and audiology-based monitoring as an important management component, particularly when preserving hearing in the better ear is a priority and immediate intervention is not mandatory.

Another notable finding of this review is the increasing relevance of multimodal and systemic management. Combined approaches—including subtotal microsurgical resection followed by radiosurgery in selected cases—have been reported at specialized centers. In parallel, bevacizumab has become an established option for progressive tumors with hearing decline, with radiographic response in roughly one third of tumors and disease stabilization in many others, albeit with toxicity and concerns about rebound growth after withdrawal [32, 45]. Brigatinib, although not part of the VS-specific evidence base included in this review, warrants mention because a 2024 phase 2 trial in progressive NF2-related schwannomatosis reported hearing improvement in 35% of eligible ears, decreased annualized growth rates across tumor types, and limited volumetric response of vestibular schwannomas overall, suggesting a potentially useful hearing-oriented systemic option that still requires longer-term and VS-specific evaluation [12].

### Influence of concomitant tumors on management

NF2-related schwannomatosis is a multi-tumor syndrome, and vestibular schwannomas rarely occur in isolation. Most patients also harbor meningiomas, non-vestibular cranial-nerve schwannomas, spinal schwannomas, and ependymomas, all of which contribute to overall morbidity and prognosis [2, 3, 6, 7]. The total tumor burden, rather than the vestibular schwannoma alone, frequently determines management priorities; in a UK national analysis of 1,192 patients, the presence of intracranial meningiomas was an independent predictor of increased mortality [47]. Coexisting tumors can therefore reorder treatment sequencing—for example, when a symptomatic meningioma or a cord-compressing spinal lesion takes precedence over a stable vestibular schwannoma—and can alter the chosen surgical corridor or contraindicate approaches that would jeopardize an already compromised cranial nerve. The cumulative lesion count also heightens concern about radiation-associated malignant progression in this tumor-predisposition syndrome, because repeated or large-field irradiation across multiple tumors increases lifetime exposure [13]. Conversely, systemic agents such as bevacizumab and brigatinib are attractive precisely because they may act on multiple tumor types simultaneously [12, 32]. Vestibular schwannoma management in NF2 should thus be planned within a whole-patient, whole-tumor framework rather than as an isolated decision, a point emphasized by both reviewers.

### Hearing rehabilitation and its impact on treatment strategy

Because progressive bilateral hearing loss is the dominant source of disability in NF2, hearing rehabilitation is not merely a downstream consequence of treatment but an active determinant of modality choice and timing [5, 8]. Two principal options exist: cochlear implantation, which is feasible when the cochlear nerve remains anatomically and functionally intact, and auditory brainstem implantation, which remains the standard when the cochlear nerve is lost or non-functional. Where the cochlear nerve is preserved, cochlear implantation generally provides better open-set speech perception than the auditory brainstem implant [48]. This consideration reframes the entire treatment algorithm: strategies that preserve the cochlear nerve—nerve-sparing microsurgery, observation, carefully selected radiosurgery, or systemic therapy used to delay growth—keep the cochlear-implant option open and prolong the window of potentially serviceable hearing [5, 26, 27, 48]. Several specialist programs in this review incorporated proactive auditory rehabilitation into the surgical plan, including auditory brainstem implantation at the time of translabyrinthine resection or cochlear implantation during nerve-sparing surgery [26, 27]. Anticipated hearing-restoration pathways should therefore be discussed before, not after, deciding how and when to treat the tumor.

### Facial, trigeminal and other rehabilitation

Facial nerve dysfunction and trigeminal morbidity substantially affect quality of life and create distinct rehabilitation needs. When facial nerve continuity is lost during resection, or function is irreversibly poor, facial reanimation can restore resting tone and voluntary movement. Hypoglossal-facial and hemihypoglossal-facial anastomosis or neurorrhaphy are the most frequently reported techniques, and outcomes are consistently better when reanimation is performed early after injury [38, 49]. Adjunctive measures—upper-eyelid loading, physiotherapy, and dedicated corneal protection—are particularly important when facial weakness coexists with trigeminal (corneal) hypoesthesia, because the combined deficit places the eye at high risk. Given the wide clinical variability across patients noted by the reviewer, these rehabilitation pathways should be anticipated during counseling and weighed when choosing both the modality and its timing, since the likelihood of facial or trigeminal injury differs systematically among microsurgery, radiosurgery, and systemic or conservative approaches.

### Sequential and salvage strategies

In practice, management is often sequential rather than a single definitive intervention, and several included studies illustrate the main pathways. Watch-and-wait was commonly followed by active treatment once growth or hearing decline occurred [28, 40]. For large tumors, planned subtotal resection followed by upfront radiosurgery to the residue achieved tumor control comparable to non-NF2 cases while sparing facial function [39]. Radiosurgery was sometimes followed by salvage microsurgery: in the Sheffield series, roughly one in five patients had undergone surgery by eight years after radiosurgery [17, 21]. Systemic therapy was used to delay or downstage local treatment. Salvage microsurgery was reported after bevacizumab failure, and rebound growth after bevacizumab withdrawal occasionally prompted surgery or irradiation [45, 46]. Importantly, a bevacizumab-free interval before surgery has been recommended to reduce intraoperative bleeding [46]. Mixed first-line strategies assigned by ear and revision surgery after prior resection were also reported [31, 38]. Taken together, these observations indicate that the initial modality should be chosen as the first step of a longitudinal, adaptable plan, with explicit attention to how each option preserves or forecloses later ones—most notably by preserving the cochlear nerve to keep cochlear implantation available and by timing systemic therapy to avoid compromising subsequent surgery.

The risk-of-bias assessment revealed that most studies were of moderate methodological quality. This reflects the inherent challenges of researching a rare disease such as NF2-related schwannomatosis, where most evidence derives from retrospective series and treatment selection is strongly influenced by tumor size, hearing status, age, and center-specific practice.

### Clinical application

**Fig. 4:**
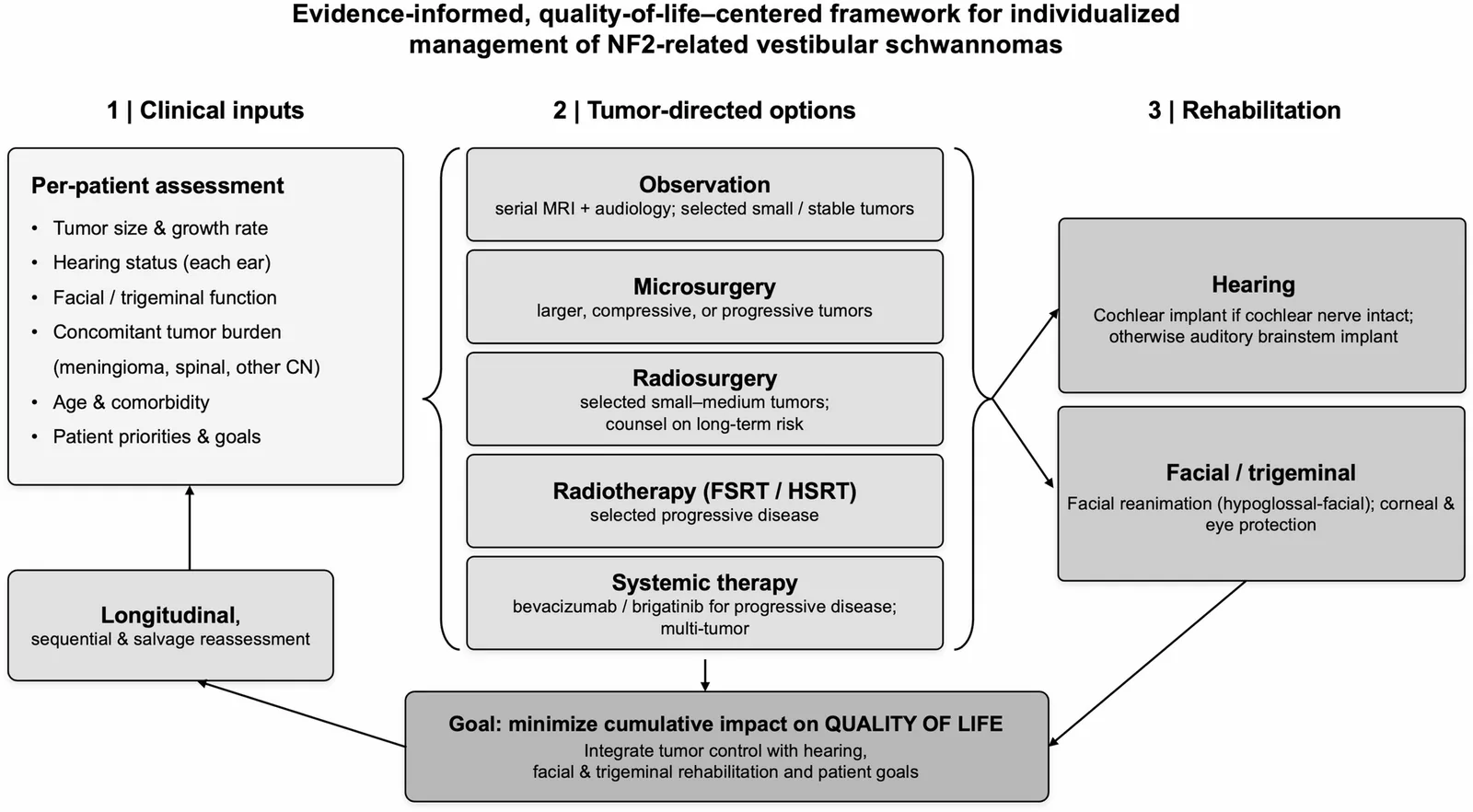
Evidence-informed, quality-of-life–centered framework for individualized management of NF2-related vestibular schwannomas. Management is structured as a continuous, three-step process: First, the per-patient assessment integrates tumor size and growth rate, hearing status in each ear, facial and trigeminal function, overall concomitant tumor burden (including meningiomas and spinal or other cranial-nerve tumors), age and comorbidity, and the patient’s priorities. Second, a tumor-directed option is selected—observation, microsurgery, radiosurgery, radiotherapy, or systemic therapy (bevacizumab or brigatinib)—recognizing that these are often used sequentially rather than as a single definitive intervention. Third, hearing rehabilitation (cochlear implantation when the cochlear nerve is intact, otherwise auditory brainstem implantation) and facial or trigeminal rehabilitation (facial reanimation and corneal protection) are planned in parallel rather than as afterthoughts. The unifying objective is to minimize the cumulative impact of the disease and its treatment on quality of life. Longitudinal, sequential, and salvage reassessment feeds back into the per-patient assessment over time. This schematic is a conceptual synthesis of the qualitative findings of this review and is not a validated treatment algorithm

The findings of this review support a patient-centered, multidisciplinary approach to NF2-associated vestibular schwannoma management. Microsurgery remains indispensable for large or compressive tumors. Observation is appropriate for selected indolent lesions. Bevacizumab and possibly brigatinib may help defer local intervention in progressive disease. Radiosurgery should be reserved for carefully selected cases after explicit counseling regarding uncertain long-term risks, including malignant progression. Across these decisions, the unifying objective is to minimize the cumulative impact of the disease and its treatment on quality of life by integrating tumor-directed control with hearing, facial, and trigeminal rehabilitation and with the patient’s own priorities. Disease-specific instruments such as the NFTI-QOL can help structure and track this assessment over time [50, 51]. An evidence-informed, quality-of-life–centered framework that brings together the clinical inputs, modality options, rehabilitation pathways, and longitudinal reassessment discussed above is summarized in Figure 4.

### Limitations

This study has limitations: (a) Most included studies were retrospective case series, introducing potential selection bias and reducing the strength of the evidence. (b) Significant heterogeneity across studies in patient populations, tumor characteristics, treatment indications, outcome definitions, and follow-up duration precluded formal meta-analysis. (c) The apparent predominance of radiosurgery in the included literature reflects publication patterns and the historical development of treatment rather than necessarily mirroring present-day practice. (d) Emerging systemic therapies such as brigatinib are not yet well represented in vestibular schwannoma-specific cohorts, so their role is evolving faster than the traditional evidence base captured here. (e) Because no minimum sample-size threshold was applied, very small case series contributed to the synthesis. Although this choice was intended to capture the full breadth of evidence for a rare disease, it increases the influence of low-precision estimates and limits the generalizability of some reported outcomes.

## Conclusion

In conclusion, the current literature indicates that management of NF2-associated vestibular schwannomas remains highly individualized, with treatment decisions balancing tumor control against long-term preservation of hearing and facial nerve function. The published literature remains weighted toward radiosurgery, but contemporary care is increasingly influenced by concerns about radiation-associated malignant progression, the selective role of observation, and expanding systemic options such as bevacizumab and brigatinib. Microsurgery remains essential for larger, symptomatic, or compressive lesions. Decisions must also account for the patient's overall tumor burden and the availability of hearing and facial rehabilitation, since the overarching goal is to reduce the cumulative impact of the disease and its treatment on quality of life by selecting and sequencing the most appropriate option for each patient. Future prospective studies with standardized outcome reporting and comparisons of modern modalities are needed to define optimal sequencing and patient selection.

## Data Availability

Data sharing is not applicable to this article, as no datasets were generated or analyzed during the current study.
